# Effects of Target Clarity on Monocular Blur Suppression: A Video Simulation of Simultaneous Vision Multifocal Lens Correction

**DOI:** 10.7759/cureus.82760

**Published:** 2025-04-22

**Authors:** Yo Iwata, Tomoya Handa

**Affiliations:** 1 School of Allied Health Sciences, Kitasato University, Sagamihara, JPN

**Keywords:** contact lens, intraocular lens, monocular blur suppression, refractive correction, simultaneous vision multifocal lens

## Abstract

Objectives: To examine the effects of target clarity on monocular blur suppression while using simultaneous vision multifocal lenses through video simulation.

Methods: Twenty healthy adults (mean age 20.8±6.1 years) were presented with circular targets at three spatial frequencies (3, 6, and 18 cpd). Gaussian blur filter simulated full refractive correction (0 pixels of blur) and two levels of residual refractive error correction (10 pixels and 20 pixels, respectively). For each target condition, a 50% transparent image with the same spatial frequency was superimposed, with its blur gradually increasing by 0.1 pixels every 0.2 s. The time until participants detected additional blur compared to the initial state was measured and analyzed.

Results: Participants perceived blur significantly earlier in the two levels of residual refractive error models (10 and 20 pixels of initial blur) compared to the full refractive correction model across all spatial frequencies (p < 0.017). Spatial frequency also influenced blur perception, with detection occurring significantly earlier at higher cpd values across initial blur conditions.

Conclusions: Residual refractive error appears to impair the function of monocular blur suppression, aggravating the perception of ghost images. Full refractive correction is therefore essential for effectively suppressing these unwanted visual artifacts.

## Introduction

As humans age, they experience hardening of the crystalline lens, resulting in decreased elasticity and reduced contractility of the ciliary muscles, ultimately leading to a decline in the eye's accommodative power. This phenomenon is known as presbyopia [1]. One method to address presbyopia is using simultaneous vision multifocal lenses, which are found in soft contact lenses [2], and intraocular lenses [3]. These lenses feature multiple refractive powers that gradually transition from distance to near vision within a single lens, allowing for clear vision across a wide range of distances [4,5]. However, images of a visual target do not solely pass through the optical zone corresponding to the appropriate refractive power. For example, when a patient wearing simultaneous multifocal contact lenses looks at a distant object, the image travels through the distance optical zone and focuses on the retina; however, the same image also traverses the near optical zone. This overlap leads to an unfocused, blurred image being projected onto the retina simultaneously, which patients may perceive as a “ghost” image [6,7]. Although the simultaneous projection of both focused and unfocused images onto the retina is an inherent characteristic of simultaneous vision, multifocal lenses, unfocused images are not necessarily perceived as ghost images. Ghost images can be suppressed to varying degrees by visual processing mechanisms such as selective attention [8,9], neural adaptation [10-12], perceptual learning [13,14], and interactions between the parvocellular and magnocellular pathways [15,16]. This visual processing is referred to as monocular blur suppression [17].

The presence of residual refractive error in simultaneous vision multifocal lenses is anticipated to compromise the efficiency of monocular blur suppression. This mechanism functions optimally when distinct sharp and blurred images overlap on the retina; however, refractive error in the lens causes even the images passing through the intended optical zone to become blurred. This diminishes the contrast between focused and unfocused images on the retina, reducing the efficiency of monocular blur suppression and making ghost images more perceptible. Complete refractive correction without residual error is therefore hypothesized to maximize ghost image suppression.

This study investigated how varying levels of visual target clarity, simulated through different spatial frequencies and degrees of blur, affect monocular blur suppression in individuals viewing through simultaneous vision multifocal lenses, using a controlled video simulation.

## Materials and methods

We initially recruited 24 healthy adults aged 20-39 years. Four participants were excluded because their astigmatism exceeded 1.50 diopters; no additional participants were excluded for ocular disorders. Consequently, 20 participants (age, 20.8±6.1 years) free from ocular diseases other than refractive errors were included in the final analysis. No restrictions were set for spherical refractive power. As this study is an initial attempt to quantify blur awareness assuming the use of multifocal lenses, and the effect size could not be estimated, the sample size was set at 20 as a pilot study. All participants had full refractive correction by subjective refraction test, and were instructed to fixate on a circular target with a visual angle of 2° displayed on a monitor at a viewing distance of 1 m. The test was conducted using monocular vision with only the right eye, with the left eye occluded. The room illuminance was set at 600-800 lx. Targets were presented using an iPad Pro 4th generation (12.9 inch, 2048×2732 resolution, 264 pixels per inch; Apple Inc., Cupertino, CA). The VideoLAN Client (VLC) media player application (App Store) was used to display the videos. Three target patterns were applied to simulate different conditions of full refractive correction (FRC) and residual refractive error correction (RREC). Each pattern was presented at three different spatial frequencies, 3, 6, and 18 cycles per degree (cpd)-resulting in nine experimental conditions. The patterns consisted of the following: square wave with no Gaussian blur filter (0.0 pixels; FRC model; 3, 6, and 18 cpd; Videos 1-3); square wave with mild Gaussian blur (10.0 pixels; RREC-10 model; 3, 6, and 18 cpd; Videos 4-6); and square wave with moderate Gaussian blur (20.0 pixels; RREC-20 model; 3, 6, and 18 cpd; Videos 7-9).

**Video 1:** Initial blur is 0 at 3 cpd.

**Video 2:** Initial blur is 0 at 6 cpd.

**Video 3:** Initial blur is 0 at 18 cpd.

**Video 4:** Initial blur is 10 at 3 cpd.

**Video 5:** Initial blur is 10 at 6 cpd.

**Video 6:** Initial blur is 10 at 18 cpd.

**Video 7:** Initial blur is 20 at 3 cpd.

**Video 8:** Initial blur is 20 at 6 cpd.

**Video 9:** Initial blur is 20 at 18 cpd.

**Figure 1:**
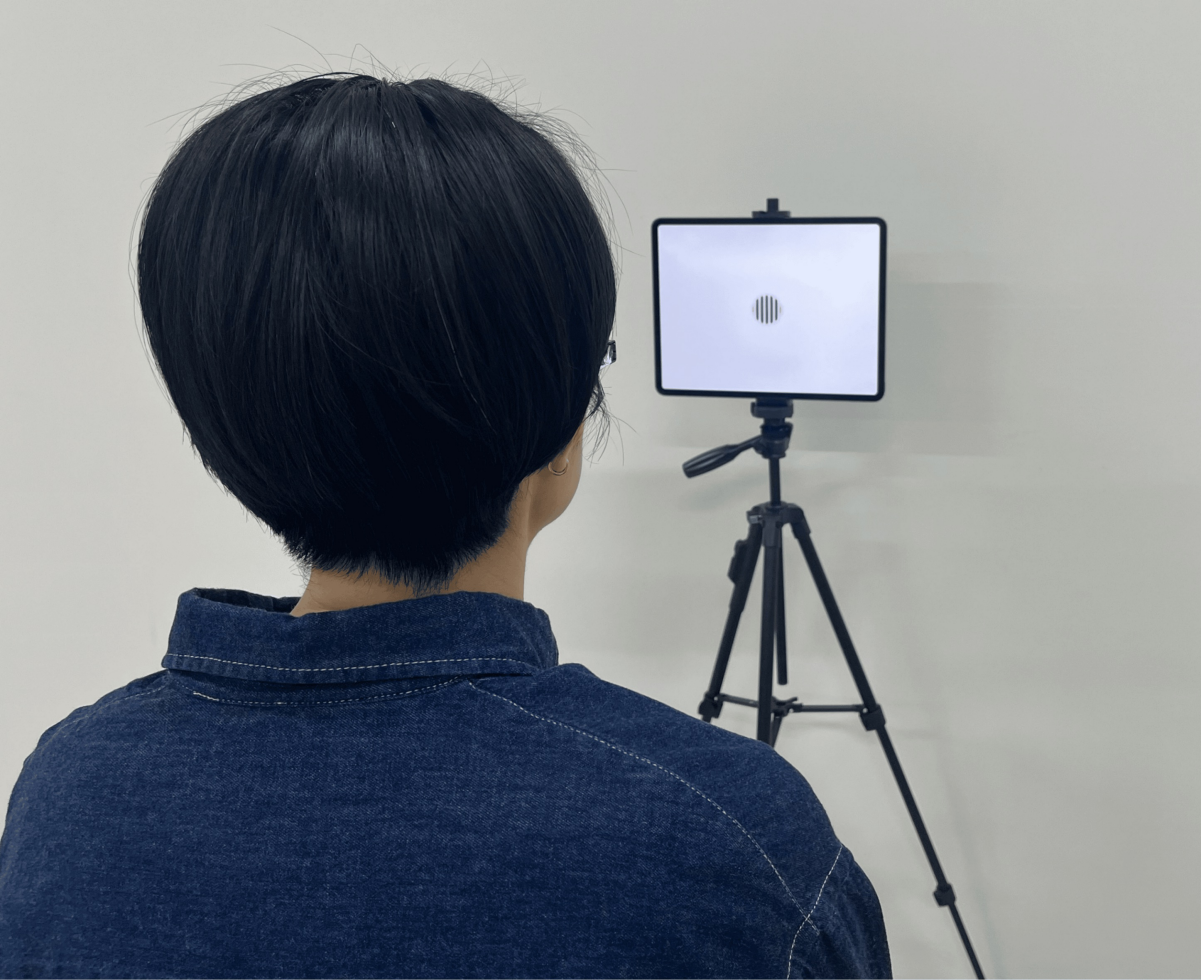
Experimental setup The subject fixates on the target.

**Figure 2:**
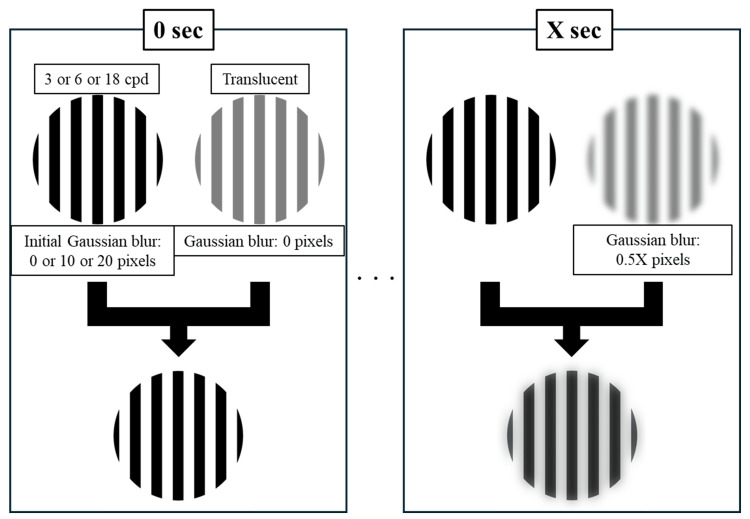
Schematic overview of the visual task. Targets consisted of nine combinations of conditions, with three levels of initial Gaussian blur (0 pixels for FRC model, 10 pixels for RREC-10 model, and 20 pixels for RREC-20 model) and three spatial frequencies (3, 6, and 18 cpd). Participants were instructed to fixate on each target with a 50% transparent image of the same spatial frequency superimposed over it. The initial Gaussian blur of this superimposed image was 0 pixels, and 0.1 pixels of blur were added every 0.2 s. The time at which participants first noticed additional blur compared to the initial state was measured.

The target presentation order was randomized. For each condition, a 50% transparent image with the same spatial frequency was superimposed over the base target. The initial Gaussian blur of this superimposed image was 0 pixels, and 0.1 pixels were added every 0.2 seconds. Prior to testing, all participants confirmed that blur was initially visible in the RREC-10 and RREC-20 targets. Participants indicated the moment they perceived additional blur compared with the initial state. Each condition was tested three times, and the average values were used for analysis. Before the tests, we provided a single training session on the tasks to ensure that participants understood the content at the point of actual measurements. There were approximately 30 s between tasks, and no other breaks were allowed. Figure 1 shows the measurement setup, and Figure 2 provides a schematic overview of the visual task. The actual target videos used are available in Videos 1-9.

The time in seconds (s) at which participants first noticed additional blur compared with the initial state was analyzed to determine in which models it was most readily perceived and, additionally, to assess the influence of spatial frequency. The Wilcoxon signed-rank test with Bonferroni adjustment was used for pairwise comparisons between models (FRC vs. RREC-10 vs. RREC-20) by spatial frequency and between spatial frequencies (3 vs. 6 vs. 18 cpd) by model. A significance level of < 5% indicated a statistically significant difference. When the Bonferroni method was used, the significance level was determined as p < 0.017.

Post hoc effect sizes were expressed as rank‑biserial correlations (rrb), interpreted as small (0.10), medium (0.30), or large (0.50). To judge whether the present sample (n = 20) was adequate, we converted each observed rrb to the corresponding Z statistic and estimated the sample size required for 80 % power at α = 0.05.

This study complied with the Declaration of Helsinki and was approved by the Japan Orthoptic Vision Society Human Sciences Ethics Committee (JOVS-25002). All procedures were performed following approved guidelines. Informed consent was obtained from all participants after explaining the study details and potential outcomes.

## Results

**Figure 3:**
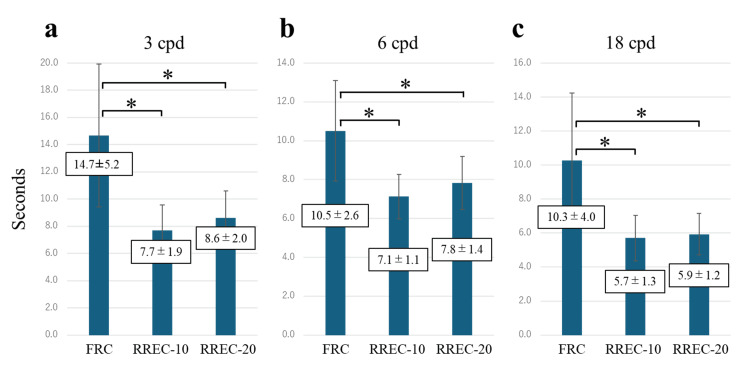
Comparison of the time at which the subjects became aware of the blurring in each spatial frequency. Bar graphs comparing the times at which additional blur was first perceived in the FRC model (initial blur: 0 pixels), RREC-10 model (10 pixels), and RREC-20 model (20 pixels) at different spatial frequencies: (a) 3 cpd (b) 6 cpd, and (c) 18 cpd. The results indicate that blur was most readily perceived in the residual refractive error models across all spatial frequencies. Data are presented as mean±SD. *p < 0.017. FRC: full refractive correction, RREC: residual refractive error correction.

For the 3 cpd target, the times to perceive blur were 14.7±5.2s for the FRC model, 7.7±1.9s for the RREC-10 model, and 8.6±2.0s for the RREC-20 model (Figure 3a). For the 6 cpd target, the times to perceive blur were 10.5±2.6s for the FRC model, 7.1±1.1s for the RREC-10 model, and 7.8±1.4s for the RREC-20 model (Figure 3b). For the 18 cpd target, the times to perceive blur were 10.3±4.0s for the FRC model, 5.7±1.3s for the RREC-10 model, and 5.9±1.2s for the RREC-20 model (Figure 3c). Significant differences were observed between the FRC model and the RREC-10 and RREC-20 models across all spatial-frequency targets, indicating that blur was most readily perceived in the residual refractive error correction models (p < 0.017).

**Figure 4:**
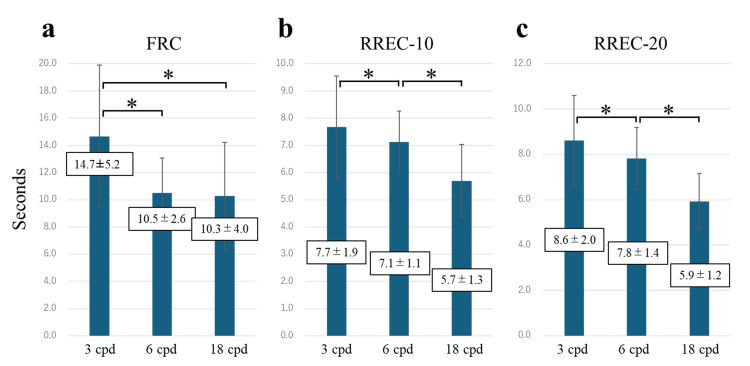
Comparison of the time at which the subjects became aware of the blurring in each model. Bar graphs comparing the times at which additional blur was first perceived at each spatial frequency in different models: (a) FRC model (initial blur: 0 pixels), (b) RREC-10 model (10 pixels), and (c) RREC-20 model (20 pixels). The results indicate that blur was more readily perceived at higher spatial frequencies. Data are presented as mean±SD. *p < 0.017. FRC: full refractive correction, RREC: residual refractive error correction.

Significant differences were also observed between spatial-frequency targets within the same model, between 3 cpd and 6 cpd, and between 3 cpd and 18 cpd in the FRC model (Figure 4a), and between 3 cpd and 6 cpd and between 6 cpd and 18 cpd in both the RREC-10 and RREC-20 models (Figures 4b, 4c), indicating that blur was more readily perceived at higher spatial frequencies (p < 0.017).

Large effect sizes were observed for all FRC-RREC comparisons. The corresponding required sample size for 80 % power was ≤ 12, indicating that our cohort of 20 participants was more than sufficient. Meanwhile, medium and small effects between the 10‑px and 20‑px RREC conditions would require 30-100 participants to achieve the same power; hence, these secondary comparisons were underpowered in the present pilot study (Table 1).

**Table 1:** Post-hoc power analysis

	r_rb_	95% CI	Magnitude	N required (80% power)	Sufficient (n=20)
3 cpd: FRC vs RREC (10px)	-0.80	-0.92 to -0.56	Large	10	Yes
3 cpd: FRC vs RREC (20px)	-0.84	-0.93 to -0.62	Large	9	Yes
3 cpd: RREC (10px) vs RREC (20px)	-0.50	-0.77 to -0.06	Medium	30	No
6 cpd: FRC vs RREC (10px)	-0.78	-0.91 to -0.51	Large	11	Yes
6 cpd: FRC vs RREC (20px)	-0.75	-0.90 to -0.46	Large	12	Yes
6 cpd: RREC (10px) vs RREC (20px)	-0.42	-0.73 to 0.03	Medium	43	No
18 cpd: FRC vs RREC (10px)	-0.83	-0.93 to -0.61	Large	9	Yes
18 cpd: FRC vs RREC (20px)	-0.84	-0.93 to -0.62	Large	9	Yes
18 cpd: RREC (10px) vs RREC (20px)	-0.28	-0.64 to 0.19	Small	98	No

## Discussion

Our study demonstrated that blur was more readily perceived in the residual refractive error models than in the full refractive correction model. When using simultaneous vision multifocal lenses with full refractive correction (without residual blur), the visual system effectively suppresses blurred images that overlap with sharp images on the retina via various processing mechanisms, including selective attention [8,9], neural adaptation [10-12], perceptual learning [13,14], and interactions between parvocellular and magnocellular pathways [15,16]. During brief exposures, as in our experiments, blur suppression appears to be primarily mediated by selective attention and pathway interactions. Specifically, selective attention prioritizes the processing of sharp images, preventing blurred images from reaching the level of conscious perception, while the parvocellular pathway, which excels at high-spatial-frequency processing, operates preferentially in the presence of sharp images. Conversely, when residual refractive error is present, these mechanisms underlying monocular blur suppression are compromised by the lack of a truly clear image, resulting in unmitigated blur and heightened perceptual salience of ghost images.

In this study, a Gaussian blur was adopted to present the same blur to the research subjects. Gaussian blur (px) and refractive power (D) cannot be converted precisely. However, the RREC-10 and RRTC-20 models exhibit only slight blurring, and we estimate that these models correspond to approximately 0.25 D and 0.50 D of refractive error, respectively.

In ophthalmological practice, visual acuity examinations are sometimes concluded once a logMAR value of approximately zero is confirmed, without necessarily detecting the absolute best visual acuity [18-20]. While this approach is sufficient when functional vision is of primary concern rather than precise refractive correction, our study indicates that when determining the optimal power of simultaneous vision multifocal lenses, inadvertent under-correction due to not detecting maximum visual acuity can result in distance images lacking clarity. This inadvertent under-correction may make it difficult for the brain to eliminate ghost images as unnecessary information, potentially reducing patient satisfaction. Full refractive correction determined via maximum visual acuity is thus essential for efficiently suppressing ghost images. Previous research has shown that when implanting multifocal intraocular lenses (IOLs), smaller deviations from targeted full refractive correction values result in higher patient satisfaction [21-23]. Additionally, multifocal intraocular lenses (IOLs) are more susceptible to the impact of power deviations from full refractive correction values on patient satisfaction than monofocal IOLs [24-26]. The implications of these findings, namely, that power deviations from full refractive correction in multifocal IOLs may prevent the elimination of ghost images, are supported by the experimental evidence obtained in the present study. Furthermore, research on multifocal contact lenses has shown that optimal neural adaptation requires retinal images to be rendered as sharply as possible [27,28]. Our study also revealed that ghost images were more readily perceived at higher spatial frequencies. This is likely because better visual acuity is demanded at higher spatial frequencies, increasing sensitivity to even slight blur.

This study had some limitations. First, all participants were young, healthy adults (age 20.8±6.1 years), whereas simultaneous vision multifocal lenses are typically prescribed to presbyopic individuals in their mid‑40s and older. Age‑related changes in ocular optics, neural adaptation, and blur‑suppression capacity might therefore limit the direct applicability of our findings to the clinical population; future studies must deliberately recruit middle‑aged and older subjects. Second, we did not collect subjective ratings of visual clarity, discomfort, or ghost‑image perception. Such patient‑reported outcomes are essential for assessing real‑world visual performance and should accompany objective measures in subsequent work. Third, blur stimuli were delivered through a video‑based simulation rather than actual multifocal lenses; factors that could alter blur perception, such as lens aberrations, tear‑film dynamics, and binocular interactions, were not reproduced.

Finally, the effect sizes were small in some comparisons, and the sample size of 20 subjects did not ensure sufficient power. In particular, in comparisons between residual refractive error models, the small effect sizes made it difficult to obtain significant differences, and the possibility of false negatives could not be completely ruled out. Future studies with larger sample sizes are warranted to better clarify these differences.

## Conclusions

By demonstrating that clear retinal images are critical for efficient monocular blur suppression, our findings highlight the importance of achieving full refractive correction when fitting simultaneous vision multifocal lenses. Moreover, the enhanced visibility of blur at higher spatial frequencies underscores the importance of fine-tuning refractive correction, particularly for tasks that demand high-resolution vision. These observations encourage a thorough refraction process and careful lens selection, ensuring that patients benefit from the full potential of multifocal lens technology. Of note, it should be considered that the subjects of this study were younger than the middle-aged and older people who typically use multifocal lenses and that this study was conducted using video simulation.
